# Association of Breast Tumour Expression of Cannabinoid Receptors CBR1 and CBR2 with Prognostic Factors and Survival in Breast Cancer Patients

**DOI:** 10.3390/jpm11090852

**Published:** 2021-08-28

**Authors:** Jessica Morin-Buote, Kaoutar Ennour-Idrissi, Éric Poirier, Julie Lemieux, Daniela Furrer, Anna Burguin, Francine Durocher, Caroline Diorio

**Affiliations:** 1 Department of Social and Preventive Medicine, Faculty of Medicine, Laval University, Quebec City, QC G1V 0A6, Canada; jessica.morin-buote.1@ulaval.ca (J.M.-B.); kaoutar.ennour-idrissi.1@ulaval.ca (K.E.-I.); 2 CHU de Québec-Université Laval Research Center, Quebec City, QC G1S 4L8, Canada; eric.poirier.med@ssss.gouv.qc.ca (É.P.); Julie.Lemieux@crchudequebec.ulaval.ca (J.L.); daniela.furrer-soliz-urrutia.1@ulaval.ca (D.F.); anna.burguin.1@ulaval.ca (A.B.); francine.durocher@crchudequebec.ulaval.ca (F.D.); 3 Université Laval Cancer Research Center, Quebec City, QC G1R 3S3, Canada; 4 Department of Molecular Biology, Medical Chemistry and Pathology, Faculty of Medicine, Laval University, Quebec City, QC G1V 0A6, Canada; 5 Department of Surgery, Faculty of Medicine, Laval University, Quebec City, QC G1V 0A6, Canada; 6 Centre des Maladies du Sein, Hôpital Saint-Sacrement, Quebec City, QC G1S 4L8, Canada; 7 Department of Medicine, Faculty of Medicine, Laval University, Quebec City, QC G1V 0A6, Canada; 8 Department of Molecular Medicine, Faculty of Medicine, Laval University, Quebec City, QC G1V 0A6, Canada

**Keywords:** breast cancer, cohort study, cannabinoid receptor, immunohistochemistry, survival, prognostic factors

## Abstract

Cannabinoid receptors (CBR) are potential therapeutic targets for breast cancer. However, the role of CBR in breast cancer survival remains poorly understood. Data from a prospective cohort of 522 women diagnosed with invasive breast cancer between 2010 and 2012 were analysed. Clinical and pathological features were retrieved from electronic medical records. CBR expression was measured by immunohistochemistry. Adjusted partial Spearman correlations and multivariate Cox models were used to estimate associations with breast cancer prognostic factors and survival, respectively. The median follow-up was 92.0 months (range 7.0–114.0). CBR expression was heterogenous in tumours. Cytoplasmic expression of CBR1 was positively correlated with lymph node invasion (r_s_ = 0.110; *p* = 0.0155) and positive status of the human epidermal growth factor receptor 2 (HER2) (r_s_ = 0.168; *p* = 0.0002), while nuclear CBR2 was negatively correlated with grade (r_s_ = −0.171; *p* = 0.0002) and positively correlated with oestrogen receptor and progesterone receptor-positive status (r_s_ = 0.173; *p* = 0.0002 and r_s_ = 0.121; *p* = 0.0084, respectively). High cytoplasmic expression of CBR2 was associated, with 13% higher locoregional and distant recurrences (HR = 1.13 [0.97–1.33]), though this association did not reach statistical significance. Although the few events occurring during follow-up may have limited the detection of significant associations, these results indicate that CBR expression in breast cancer deserves further investigation.

## 1. Introduction

Cannabinoids receptors (CBR) have been suggested as potential therapeutic targets for breast cancer [1,2,3,4,5,6]. These receptors are part of the family of membrane receptor G protein-coupled receptors and have been shown to be involved in signaling pathways regulating proliferation, cancer cell survival, angiogenesis as well as tumour invasion [7,8]. Thus far, two subtypes of CBR have been identified: CBR1, which is found in the brain and in certain nerve endings but also in reproductive organs, vascular endothelium and eyes [9] and CBR2, which is expressed mainly in the immune system, membranes of immune and tumour cells, spleen, bones, tonsils and thyroid gland [4,10,11].

Previous studies have shown that CBR1 and CBR2 are involved in reducing the progression of tumours in animal models and have antitumoral action in vivo [12,13,14,15], mainly by inducing apoptosis and cell cycle arrest [2]. Indeed, high CBR1 and/or CBR2 expression has been shown to be associated with survival in prostate cancer [16,17,18] and colorectal cancer [19,20]. However, divergent results regarding the direction of these associations were reported [21,22]. These differences might be explained by methodological biases (selection bias, lack of clinical and pathological information, small sample size). Regarding breast cancer, evidence suggests that CBR alters the migration and invasion of estrogen receptor (ER) and progesterone receptor (PR)—positive breast cancer cells in culture [23]. Likewise, inhibition of cell proliferation has been observed in other breast cancer cell lines [4,24,25,26]. Thus far, a single epidemiological study has evaluated the relationship between CBR2 expression and breast cancer survival and reported that high CBR2 expression was associated with decreased overall survival, increased local recurrence and development of distant metastases [27]. However, possible confounding factors were not considered in the study, which could have led to biased results and CBR1 has not been investigated. 

Therefore, in the present study, we aim to evaluate CBR1 and CBR2 expression in breast tumours and their association with breast cancer prognostic factors and survival.

## 2. Materials and Methods

### 2.1. Study Design and Population

A longitudinal study was carried out at the Centre des Maladies du Sein (CMS) of Quebec City, Canada, a reference centre for breast diseases. Women diagnosed with invasive breast cancers were recruited prospectively between 1 December 2010 and 30 April 2012. Detailed information on recruitment was described elsewhere [28]. Women were included if they (1) had a mastectomy or a segmental mastectomy (2) agreed to donate tissue specimens to the biobank of the CMS; (3) had their clinical follow-up at the CMS. Follow-up data were collected until December 2020. The end date of the study period was defined as the date of last contact (the last CMS appointment, last phone communication documented or last clinical reports). The present study was approved by the research ethics of the CHU de Québec—Laval University Research Center (DR-002-938), and all patients gave their written consent.

### 2.2. Data Collection

All patient characteristics were collected at diagnosis and included age at diagnosis (years), parity (yes vs. no), first-degree family history of breast cancer (yes vs. no), personal history of breast cancer (yes vs. no), smoking status (former or current smokers vs. never smoked), alcohol consumption (yes vs. no), menopausal status (premenopausal vs. postmenopausal) and body mass index (BMI, kg/m^2^, calculated from self-reported weight and height). All clinical and pathological characteristics were collected by trained nurses from electronic patient records (EPR) and included: tumour size (mm), lymph node involvement (number), grade (1 vs. 2 vs. 3), stage (I vs. II vs. III vs. IV), ER/PR receptor status (negative vs. positive), human epidermal growth factor receptor 2 (HER2) status (negative vs. positive), surgery (mastectomy vs. partial mastectomy), endocrine therapy (yes vs. no), chemotherapy (yes vs. no), radiotherapy (yes vs. no) and follow-up events (occurring at least 1 year after diagnosis)—locoregional recurrence (yes vs. no), distant recurrence (yes vs. no), second invasive cancer (yes vs. no) or death (yes or no). Locoregional and distant recurrence was defined as a recurrence symptom confirmed by clinical examination, positive cytology or biopsy or a positive medical imaging result [29].

### 2.3. Assessment of CBR Expression

All mastectomy specimens were collected during surgery, fixed with 10% buffered formalin within 30 min, then embedded in paraffin and cut into 4 µm tissue sections stained with hematoxylin and eosin (H&E). Invasive breast cancers were confirmed by histological examination by senior pathologists and recorded in pathology reports. For each woman, a representative tumour block was selected by a pathologist blinded to clinical data. Subsequently, 4 cores (0.6 mm in diameter each) of tumour tissue were extracted from formalin-fixed paraffin-embedded (FFPE) tissue blocks and used to build a tissue micro array (TMA). The reliability of using TMAs in histological analysis has already been demonstrated in breast cancer [30].

CBR1 and CBR2 expression were evaluated by immunohistochemistry (IHC). Deparaffinization and rehydration were, respectively, performed in toluene and ethanol baths according to standard protocols. Tris-EGTA buffer (pH9) was used for antigen retrieval (30 min, 95.6 °C). Endogenous peroxidase and non-specific antibody binding were blocked respectively by 0.3% hydrogen peroxide (H_2_O_2_) (diluted in methanol) and Super Block (IDetect). Tissues were then incubated with the primary antibody for CBR1 (CB1-Rb-Af380 [RRID: AB_2571591] from Frontier Institute) or CBR2 (CB2 receptor polyclonal antibody [101550] from Cayman Chemical) overnight at 4°C in a wet chamber, and then with the secondary antibody (Advance HRP Link, Dako) during 30 min. Tissues were treated with 3, 3′-diaminobenzidine and counterstained with Mayer’s hematoxylin. Breast cancer cell lines were present on our cohort blocks (MCF-7, SK-BR3, MDA-MB-175 and MDA-MB-231), and control blocks known to express positive and negative control tissues and cell lines for CBR1 and CBR2 were also used before proceeding with the IHC. Stained slides were scanned with the NanoZoomer 2.0-HT scanner (Hamamatsu, Bridgewater, NJ, USA).

**Figure 1:**
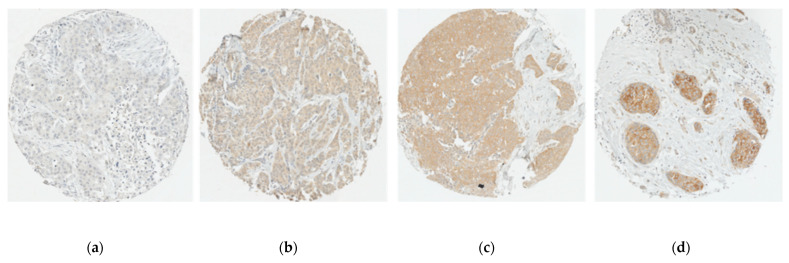
Representative immunostaining of tumour cores for CBR1: Cannabinoid receptor 1 expression intensity scores; (**a**) 0 (none); (**b**) 1 (weak); (**c**) 2 (moderate); (**d**) 3 (strong). Scale: 10X.

**Figure 2:**
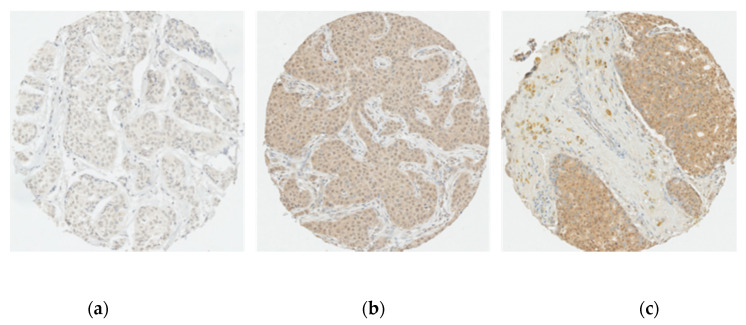
Representative immunostaining of tumour cores for CBR2: Cannabinoid receptor 2 expression intensity scores; (**a**) 0 (none); (**b**) 1 (weak cytoplasmic and nuclear staining); (**c**) 2 (moderate cytoplasmic and nuclear staining). Scale: 10X.

Samples were interpreted if the number of tumour cells was greater than 20 cancer cells on each core. Staining was scored semi-quantitatively according to intensity and proportion. Staining intensity was visually evaluated and scored either 0 (absent), 1 (weak staining), 2 (moderate staining) or 3 (strong staining). Proportion of stained cells were scored 0 (<1%), 1 (1–24%), 2 (25–50%), 3 (51–75%), 4 (>75%) [31]. A composite score (CS) was calculated by multiplying the intensity and percentage scores for each core. For each patient, the mean CS was generated from 1 to 4 cores judged interpretable. The CS was then dichotomized into low and high expression, using the median as the cut-off [32,33]. Immunostaining assessment was performed twice by two independent readers for 10% randomly selected patients, and substantial agreements were observed (kappas = 0.72, 0.80 and 0.72 for CBR1, CBR2 nuclear and CBR2 cytoplasmic, respectively). Figure 1 and Figure 2 show representative immunostaining of tumour cores.

### 2.4. Survival Outcome

Overall survival (OS) was defined as the time between breast cancer diagnosis and death from any cause. Recurrence-free survival (RFS) was defined as the time between breast cancer diagnosis and first ipsilateral locoregional recurrence or first distant recurrence, whichever occurred first. Event-free survival (EFS) was defined as the time between breast cancer diagnosis to first ipsilateral locoregional recurrence, or first distant recurrence, or contralateral invasive breast cancer, or the development of any second invasive cancer or death from any cause, whichever occurred first.

### 2.5. Statistical Analyses

Means with standard deviations and medians were reported for continuous data. Categorical variables were reported as frequencies and percentages. CBR expression was treated as a continuous variable for all analyses. Adjustment variables were selected a priori based on the literature review. The decision to include or not each factor as an adjustment variable for a specific analysis was based on the disjunctive causes criterion [34].

Correlations between breast cancer prognostic factors and CBR expression were estimated using Spearman rank-based correlations adjusted for age at diagnosis (years), menopausal status (premenopausal vs. postmenopausal), smoking status (former or current smokers vs. never smoked), alcohol consumption (yes vs. no), personal history of breast cancer (yes vs. no) and chemotherapy prior to surgery (yes vs. no). Further adjustment for BMI (kg/m^2^) was considered in a sensitivity analysis.

Univariate and multivariate Cox proportional-hazard models were used to estimate hazard ratios (HRs) and 95% confidence intervals (CIs) for OS, RFS and EFS. Participants who did not experience any event and were alive at the date of the last contact were censored at the time of the last contact. The proportional hazards assumption was assessed for each covariate with Schoenfeld residual plots. Interaction terms with time were added for variables that did not meet this hypothesis. Partially adjusted models included age at diagnosis (years), menopausal status (premenopausal vs. postmenopausal), first-degree family history of breast cancer (yes vs. no), smoking status (former or current smokers vs. never smoked), alcohol consumption (yes vs. no), personal history of breast cancer (yes vs. no), chemotherapy prior to surgery (yes vs. no) and endocrine therapy prior to surgery (yes vs. no). Fully adjusted models were further adjusted for years from diagnosis (years), trastuzumab treatment (yes vs. no), endocrine therapy (yes vs. no), radiotherapy (yes vs. no) and type of surgery (mastectomy vs. partial mastectomy). All models were further adjusted for prognostic factors—tumour size (mm), lymph node involvement (number), grade (1 vs. 2 vs. 3), stage (I vs. II vs. III vs. IV), ER/PR receptor status (negative vs. positive) and HER2 status (negative vs. positive)—in sensitivity analyses. These factors were potentially intermediate factors between CBR expression and survival.

All statistical tests were two-sided with significance *p*-value of 5%. Analyses were conducted using SAS 9.4 software (SAS Institute Inc., Cary, NC, USA).

## 3. Results

### 3.1. Study Population

Of the 522 women meeting the inclusion criteria, 489 (93.7%) and 475 (91.0%) had interpretable CBR1 and CBR2 samples, respectively. The median time between diagnosis and surgery was 42 days, and the median follow-up time was 92.0 months (mean 84.7 ± 20.7, range 7.0–114.0). The median age at diagnosis was 61.0 years (mean 61.2 ± 12.6 years, range 24.0–92.0). Most patients were postmenopausal, and about half of them had a family history of breast cancer. Most of them had an invasive ductal carcinoma stage I or II. During the follow-up period, 74 (14.2%) women developed a recurrence, and 86 (16.5%) deaths occurred. Characteristics of the study population are described in Table 1.

CBR1 immunoreactivity was observed in the cytoplasm and was heterogeneous within and between evaluated tumours. High expression of CBR1 was observed in 254 (51.9%) patients. Women with high CBR1 tumour expressions were slightly more likely to have positive lymph nodes, stage III and IV disease, HER2 positive status and to have received adjuvant chemotherapy, hormone therapy and trastuzumab therapy (Table 1).

CBR2 immunoreactivity was observed in the cytoplasm and in the nucleus and was heterogeneous within and between evaluated tumours. High CBR2 expression in the cytoplasm was observed in 264 (55.6%) patients. These patients were slightly more likely to have positive lymph nodes, stage IV disease, positive HER2 status and to have received adjuvant chemotherapy and trastuzumab therapy (Table 1). High CBR2 expression in the nucleus was observed in 269 (56.6%) patients. These patients were less likely to have grade 3 tumours, negative ER status and more likely to have received adjuvant hormone therapy (Table 1).

### 3.2. CBR Expression and Breast Cancer Prognostic Factors

CBR1 expression was positively correlated with positive lymph nodes (r_s_ = 0.107, *p* = 0.0194) and HER2 positive status (r_s_ = 0.165, *p* = 0.0003). No correlation was observed between CBR1 and histological type, tumour size, grade, disease stage and ER/PR status.

No correlation was observed between CBR2 cytoplasmic expression and histological type, tumour size, positive lymph nodes, grade, disease stage, ER/PR status and HER2 status.

CBR2 nuclear expression was positively correlated with histological type (r_s_ = 0.147, *p* = 0.0014) and ER/PR status (r_s_ = 0.173, *p* = 0.0002, r_s_ = 0.121, *p* = 0.0087) and negatively correlated with tumour grade (r_s_ = −0.170, *p* = 0.0002). No correlation was observed between CBR2 nuclear expression and tumour size, positive lymph nodes, disease stage and HER2 status (Table 2).

### 3.3. CBR Expression and Breast Cancer Survival

Associations of CBR expression and survival are shown in Table 3. 

Patients with high CBR1 expression had a median overall survival of 93.0 months (mean 86.3 ± 19.6, range 11.0–114.0). No association was observed between CBR1 expression and survival in our cohort.

Patients with high CBR2 cytoplasmic expression had a median overall survival of 92.0 months (mean 84.4 ± 21.2, range 7.0–114.0). Each one unit increase of cytoplasmic expression of CBR2 was associated with 13% higher locoregional and distant recurrences, though this association did not reach statistical significance (HR = 1.13 [0.97–1.33]).

Patients with high CBR2 nuclear expression had a median overall survival of 92.0 months (mean 84.7 ± 20.9, range 7.0–114.0). No association was observed between nuclear expression of CBR2 and survival outcomes.

Subsequent sensitivity analyses with adjustment for prognostic factors yielded similar results (Supplementary Table S1).

## 4. Discussion

In the present study, we aimed to evaluate CBR1 and CBR2 expression in breast tumours and its association with breast cancer prognostic factors and survival. CBR1 and CBR2 expression in breast tumours were detectable by IHC. CBR1 had a cytoplasmic expression, whereas CBR2 had both a cytoplasmic and nuclear expression. While CBR1 expression was positively correlated with lymph node metastases and HER2 status, nuclear CBR2 expression was positively correlated with histologic type, ER/PR positive status and negatively correlated with tumour grade. Although not statistically significant, cytoplasmic CBR2 expression was associated with higher locoregional and distant recurrences.

Our results indicate that CBR1 and CBR2 may have different involvement in breast cancer progression. In fact, CBR1 expression was correlated with poor prognostic markers while nuclear expression of CBR2 was correlated with good prognostic markers. In addition, the cellular compartment harbouring CBR expression may have a different functional significance, as we observed that nuclear expression of CBR2 had an opposite direction of association with prognosis than cytoplasmic expression of CBR2. In comparison, other studies observed in prostate cancer that CBR1 expression was positively correlated with the epidermal growth factor receptor (EGFR) (r_s_ = 0.316, *p* < 0.001) [18]. Other similarities have been observed where CBR1 expression was positively correlated with Gleason score (r_s_ = 0.21, *p* < 0.001), tumour stage (r_s_ = 0.11, *p* < 0.05), percentage of the specimen that contains tumours (r_s_ = 0.17, *p* = 0.01) and EGFR (r_s_ = 0.18, *p* = 0.01) [16]. Thus, making CBR1 an indicator of poor prognosis. While no previous study has examined the expression of CBR1 in breast cancer, the single previous study that evaluated CBR2 expression in breast cancer reported that high CBR2 expression in HER2 positive tumours was associated with poor prognosis [27]. However, in this study, only staining intensity was recorded (proportion of positive cells was not considered), and no distinction between nuclear or cytoplasmic expression was reported. Furthermore, the results from this study might have suffered from confounding biases (no adjustment for any confounders) and selection biases (analyses restricted to HER2 positive breast cancers). In fact, they reported that the expression pattern of CBR2 varies by breast cancer subtypes and that the majority of positive tumours are HER2 positive. This indicates that CBR2 might be linked to HER2 receptor expression. Nevertheless, our observation of higher recurrences in tumours with a high cytoplasmic expression of CBR2 is consistent with their results.

Our study has several strengths. Our large sample size obtained from consecutive inclusion of eligible patients is representative of breast cancer patients, as reflected by the distribution of patients’ characteristics, which reduced the risk of selection bias. Of note, our study population includes all subtypes of breast cancer, in the same proportion as in the target population of breast cancer patients. Furthermore, we used prospectively collected data from a reliable clinical database, such as more than 90% of our eligible population was included in the analyses, also preventing selection bias due to missing values. We used data collected from medical records by trained nurses and measures of CBR expression evaluated twice by two independent readers blinded to patients’ characteristics and outcomes, thus preventing the risk of information bias. Finally, we used a robust method for selecting and adjusting for potential confounders to minimize confounding bias while preventing collider bias and adjustment for potential intermediate factors. All of which ensure the internal validity of our analyses.

The main limitation to our study was the few events that occurred during the follow-up, despite the large sample size and the long follow-up period. Indeed, the proportion of patients with locoregional recurrence, distant recurrence and death may have limited our statistical power to detect genuine associations. We did not adjust for key variables such as economic status and cannabis consumption as these variables were not collected and thus were not available in our database. In fact, economic status has been identified as a predictor of survival in breast cancer patients [35,36,37] and has been shown to be associated with cannabis consumption [38,39]. However, regular cannabis consumption rates in Canada are very low [40], and the link between cannabis consumption and CBR expression is yet to be established. Although these limitations should be addressed in future studies, our findings make an important contribution to the emerging body of knowledge on the role of CBR in breast cancer survival.

## 5. Conclusions

This is the first study to evaluate both CBR1 and CBR2 expression in association with breast cancer prognostic factors and survival. We evaluated CBR expression in breast cancer tissue. CBR1 cytoplasmic expression seemed to be associated with poor prognostic factors, while CBR2 nuclear expression seemed to be associated with better prognostic factors. The cytoplasmic expression of CBR2 may be involved in the occurrence of locoregional and distant recurrences. Although the few events occurring during the follow-up may have limited the detection of significant associations, these results indicate that CBR expression in breast cancer needs further investigation.

## Figures and Tables

**Table 1:** Patients characteristics.

Characteristics *	All (*n* = 522)	CBR1 (*n* = 489)		CBR2 in Cytoplasm (*n* = 475)		CBR2 in Nuclear (*n* = 475)	
		Low * (*n* = 235)	High * (*n* = 254)	Low * (*n* = 211)	High * (*n* = 264)	Low * (*n* = 206)	High * (*n* = 269)
Age (years)							
Mean ± SD	61.2 ± 12.6	62.3 ± 12.6	60.4 ± 12.99	62.9 ± 12.6	60.0 ± 12.8	61.9 ± 13.3	60.8 ± 12.3
Median [range]	61.0 [24.0–92.0]	63.0 [32.0–91.0]	61.0 [24.0–92.0]	64.0 [24.0–91.0]	61.0 [25.0–92.0]	63.0 [24.0–91.0]	61.0 [25.0–92.0]
Postmenopausal	399 (76.4%)	186 (79.2%)	189 (74.4%)	168 (79.6%)	194 (73.5%)	155 (75.2%)	207 (77.0%)
Familial history of breast cancer (yes)	245 (46.9%)	110 (46.8%)	123 (48.4%)	97 (45.9%)	125 (47.4%)	96 (46.6%)	126 (46.8%)
Ever smokers	254 (48.7%)	119 (50.6%)	118 (46.5%)	112 (53.1%)	120 (45.5%)	95 (46.1%)	137 (50.9%)
Alcohol consumption (yes)	337 (64.6%)	147 (62.6%)	168 (66.1%)	128 (60.7%)	179 (67.8%)	131 (63.6%)	176 (65.4%)
Body mass index (kg/m^2^)							
Mean ± SD	26.3 ± 5.2	25.8 ± 5.2	26.6 ± 5.3	26.1 ± 5.0	26.4 ± 5.5	26.3 ± 5.2	26.2 ± 5.3
Median [range]	25.5 [15.2–45.9]	24.6 [16.3–44.1]	25.6 [15.2–45.9]	25.0 [17.7–44.1]	25.5 [15.2–45.9]	25.6 [16.3–44.6]	25.3 [15.2–45.9]
Personal history of breast cancer (yes)	63 (12.1%)	25 (10.6%)	32 (12.6%)	26 (12.3%)	29 (11.0%)	23 (11.2%)	32 (11.9%)
Histologic type							
Ductal, invasive	451 (86.4%)	204 (86.8%)	220 (86.6%)	180 (85.3%)	235 (89.0%)	189 (91.8%)	226 (84.0%)
Lobular, invasive	56 (10.7%)	27 (11.5%)	24 (9.5%)	24 (11.4%)	23 (8.7%)	12 (5.8%)	35 (13.0%)
Mixed ductal and lobular, invasive	15 (2.9%)	4 (1.7%)	10 (3.9%)	7 (3.3%)	6 (2.3%)	5 (2.4%)	8 (3.0%)
Tumour grade							
1	86 (16.5%)	39 (16.6%)	38 (15.0%)	30 (14.2%)	42 (18.9%)	23 (11.2%)	49 (18.2%)
2	258 (49.4%)	112 (47.7%)	130 (51.2%)	119 (56.4%)	116 (43.9%)	90 (43.7%)	145 (53.9%)
3	178 (34.1%)	84 (35.7%)	86 (33.9%)	62 (29.4%)	106 (40.2%)	93 (45.2%)	75 (27.9%)
Tumour size							
≤2 cm	293 (56.1%)	130 (55.3%)	140 (55.1%)	114 (54.0%)	148 (56.1%)	110 (53.4%)	152 (56.5%)
>2 and ≤5 cm	209 (40.0%)	101 (43.0%)	99 (39.0%)	89 (42.2%)	106 (40.2%)	93 (45.2%)	102 (37.9%)
>5 cm	20 (3.8%)	4 (1.7%)	15 (5.9%)	8 (3.8%)	10 (3.8%)	3 (1.5%)	15 (5.6%)
Positive lymph nodes							
0	312 (59.8%)	153 (65.1%)	137 (53.9%)	135 (64.0%)	146 (55.3%)	125 (60.7%)	156 (58.0%)
1–3	146 (28.0%)	60 (25.5%)	78 (30.7%)	55 (26.1%)	80 (30.3%)	59 (28.6%)	76 (28.3%)
4–9	42 (8.0%)	17 (7.2%)	22 (8.7%)	14 (6.6%)	26 (9.8%)	18 (8.7%)	22 (8.2%)
≥10	22 (4.2%)	5 (2.1%)	17 (6.7%)	7 (3.3%)	12 (4.6%)	4 (1.9%)	15 (5.6%)
Disease stage							
I	203 (38.9%)	96 (40.9%)	91 (35.8%)	84 (39.8%)	97 (36.7%)	76 (36.9%)	105 (39.0%)
II	241 (46.2%)	110 (46.8%)	119 (46.9%)	101 (47.9%)	122 (46.2%)	104 (50.5%)	119 (44.2%)
III	71 (13.6%)	28 (11.9%)	38 (18.0%)	25 (11.9%)	39 (14.8%)	24 (11.7%)	40 (14.9%)
IV	7 (1.3%)	1 (0.4%)	6 (2.4%)	1 (0.5%)	6 (2.3%)	2 (1.0%)	5 (1.9%)
ER status							
Negative	64 (12.3%)	29 (12.3%)	30 (11.8%)	35 (16.6%)	25 (9.5%)	37 (18.0%)	23 (8.6%)
Positive	458 (87.7%)	206 (87.7%)	224 (88.2%)	176 (83.4%)	239 (90.5%)	169 (82.0%)	246 (91.5%)
PR status							
Negative	108 (20.7%)	46 (19.6%)	52 (20.5%)	51 (24.1%)	47 (17.8%)	53 (25.7%)	45 (16.7%)
Positive	414 (79.3%)	189 (80.4%)	202 (79.5%)	160 (75.8%)	217 (82.2%)	153 (74.3%)	224 (83.3%)
HER2 status							
Negative	455 (87.2%)	215 (91.5%)	210 (82.7%)	191 (90.5%)	222 (84.1%)	180 (87.4%)	233 (86.6%)
Positive	67 (12.8%)	20 (8.5%)	44 (17.3%)	20 (9.5%)	42 (15.9%)	26 (12.6%)	36 (13.4%)
Surgery							
Partial	384 (73.6%)	173 (73.6%)	187 (73.6%)	156 (73.9%)	195 (73.9%)	154 (74.8%)	197 (73.2%)
Total	138 (26.4%)	62 (26.4%)	67 (26.4%)	55 (26.1%)	69 (26.1%)	52 (25.2%)	72 (26.8%)
Chemotherapy prior to surgery (yes)	6 (1.2%)	1 (0.4%)	2 (0.8%)	1 (0.5%)	2 (0.8%)	1 (0.5%)	2 (0.7%)
Hormone therapy prior to surgery (yes)	6 (1.2%)	3 (1.3%)	3 (1.2%)	4 (1.9%)	2 (0.8%)	1 (0.5%)	5 (1.9%)
Chemotherapy after surgery (yes)	263 (50.4%)	108 (46.0%)	138 (54.3%)	94 (44.6%)	146 (55.3%)	106 (51.5%)	134 (49.8%)
Radiotherapy after surgery (yes)	414 (79.3%)	184 (78.3%)	204 (80.3%)	164 (77.7%)	212 (80.3%)	163 (79.1%)	213 (79.2%)
Hormone therapy after surgery (yes)	433 (83.0%)	189 (80.4%)	217 (85.4%)	164 (77.7%)	229 (86.7%)	157 (76.2%)	236 (87.7%)
Trastuzumab after surgery (yes)	59 (11.3%)	20 (8.4%)	36 (14.2%)	15 (7.1%)	39 (14.8%)	23 (11.2%)	31 (11.5%)
Follow-up (months)							
Mean ± SD	84.7 ± 20.7	83.0 ± 21.7	86.3 ± 19.6	84.6 ± 20.8	84.4 ± 21.2	84.7 ± 21.2	84.7 ± 20.9
Median [range]	92.0 [7.0–114.0]	91.0 [7.0–112.0]	93.0 [11.0–114]	93.0 [7.0–112.0]	92.0 [7.0–114.0]	92.0 [14.0–112.0]	92.0 [7.0–114.0]
Survival outcomes							
Deaths	86 (16.5%)	41 (17.5%)	40 (15.8%)	39 (18.5%)	43 (16.3%)	42 (20.4%)	40 (14.9%)
Recurrences ^§^	74 (14.2%)	31 (13.2%)	36 (14.2%)	24 (11.4%)	44 (16.7%)	31 (15.1%)	37 (13.8%)
Events ^†^	143 (27.4%)	65 (27.7%)	69 (27.2%)	60 (28.4%)	74 (28.0%)	65 (31.6%)	69 (25.7%)

CBR1: cannabinoid receptor 1; CBR2: cannabinoid receptor 2; *n* = number; mean ± standard deviation; SD: standard deviation; ER: estrogen receptor; PR: progesterone receptor; HER2: human epidermal growth factor receptor 2; * Dichotomized at the median; ^§^ Locoregional and distant recurrences; ^†^ deaths, recurrences, any second cancer.

**Table 2:** Correlations between CBR1 and CBR2 expression and breast cancer prognostic factors.

	CBR1 (*n* = 489)			CBR2 in Cytoplasm (*n* = 475)			CBR2 in Nuclear (*n* = 475)		
	Unadjusted	Adjusted *	Fully Adjusted ^§^	Unadjusted	Adjusted *	Fully Adjusted ^§^	Unadjusted	Adjusted *	Fully Adjusted ^§^
Histologic type									
r_s_	−0.009	0.004	0.006	−0.022	−0.004	−0.002	0.144	0.148	0.147
*p*-value	0.8451	0.9316	0.9045	0.6399	0.9301	0.9602	0.0016	0.0013	0.0014
Tumour size									
r_s_	0.017	0.025	0.011	0.030	0.046	0.032	0.013	0.023	0.030
*p*-value	0.7148	0.5795	0.8074	0.5158	0.3207	0.4843	0.7721	0.6163	0.5163
Positive lymph nodes									
r_s_	0.116	0.110	0.107	0.072	0.063	0.060	0.060	0.063	0.064
*p*-value	0.0103	0.0155	0.0194	0.1180	0.1767	0.1978	0.2158	0.1770	0.1676
Tumour grade									
r_s_	0.003	−0.006	−0.014	0.052	0.043	0.036	−0.170	−0.171	−0.170
*p*-value	0.9451	0.9008	0.7632	0.2572	0.3577	0.4420	0.0002	0.0002	0.0002
Disease stage									
r_s_	0.073	0.077	0.067	0.050	0.055	0.046	0.020	0.025	0.030
*p*-value	0.1083	0.0898	0.1444	0.2812	0.2310	0.3242	0.6603	0.5929	0.5227
ER status									
r_s_	0.028	0.021	0.022	0.096	0.089	0.090	0.172	0.173	0.173
*p*-value	0.5448	0.6405	0.6336	0.0358	0.0546	0.0528	0.0002	0.0002	0.0002
PR status									
r_s_	−0.023	−0.031	−0.029	0.033	0.025	0.026	0.119	0.121	0.121
*p*-value	0.6081	0.5027	0.5287	0.4704	0.5963	0.5755	0.0096	0.0084	0.0087
HER2 status									
r_s_	0.176	0.168	0.165	0.086	0.080	0.076	0.031	0.032	0.034
*p*-value	<0.0001	0.0002	0.0003	0.0623	0.0848	0.0990	0.4989	0.4915	0.4695

CBR1: cannabinoid receptor 1; CBR2: cannabinoid receptor 2; ER: estrogen receptor; PR: progesterone receptor; HER2: human epidermal growth factor receptor 2; *n*= number; r_s_: Spearman correlation coefficient; * Adjusted for age at diagnosis, menopausal status, smoking status, alcohol consumption, personal history of breast cancer and prior chemotherapy; ^§^ Further adjusted for body mass index.

**Table 3:** Hazard ratios for the association between CBR expression and survival in breast cancer patients.

CBR	Events/Total	Crude HR (95% CI)	*p*-Value	Adjusted ^§^ HR (95% CI)	*p*-Value	Fully Adjusted ^†^ HR (95% CI)	*p*-Value
Overall survival							
CBR1	81/489	0.98 [0.88–1.09]	0.70	1.01 [0.90–1.13]	0.86	1.02 [0.91–1.14]	0.74
CBR2 cytoplasmic	82/475	0.95 [0.82–1.09]	0.46	1.03 [0.88–1.20]	0.74	1.02 [0.87–1.19]	0.84
CBR2 nuclear	82/475	0.99 [0.90–1.09]	0.76	1.01 [0.90–1.11]	0.91	1.01 [0.92–1.12]	0.81
Recurrence-free survival							
CBR1	67/489	1.04 [0.93–1.17]	0.49	1.02 [0.92–1.14]	0.71	1.04 [0.93–1.18]	0.49
CBR2 cytoplasmic	68/475	0.96 [0.83–1.11]	0.59	1.13 [0.97–1.33]	0.13	1.09 [0.93–1.28]	0.28
CBR2 nuclear	68/475	0.99 [0.90–1.09]	0.77	1.01 [0.90–1.13]	0.86	1.01 [0.90–1.13]	0.86
Event-free survival							
CBR1	134/489	1.00 [0.92–1.09]	0.97	1.01 [0.93–1.10]	0.75	1.01 [0.93–1.10]	0.81
CBR2 cytoplasmic	134/475	0.98 [0.87–1.10]	0.71	1.01 [0.90–1.14]	0.81	1.01 [0.90–1.14]	0.82
CBR2 nuclear	134/475	0.98 [0.91–1.06]	0.69	1.00 [1.00–1.00]	0.22	1.00 [1.00–1.00]	0.25

CBR: cannabinoid receptor; CBR1: cannabinoid receptor 1; CBR2: cannabinoid receptor 2; HR: hazard ratio; CI: confident interval; ^§^ Models included age, menopausal status, family history of breast cancer, smoking status, alcohol consumption, personal history of breast cancer, neoadjuvant chemotherapy, neoadjuvant endocrine therapy; ^†^ Models included age, menopausal status, family history of breast cancer, smoking status, alcohol consumption, personal history of breast cancer, neoadjuvant chemotherapy, neoadjuvant endocrine therapy, year diagnosis, adjuvant chemotherapy, adjuvant radiotherapy, adjuvant endocrine therapy, anti-HER2 therapy, type of surgery.

## Data Availability

The data that support the findings of this study are available upon reasonable request from the corresponding author [C.D.]. The data are not publicly available due to legal restrictions to respect research participant privacy and consent.

## Supplementary Materials

The following are available online at https://www.mdpi.com/article/10.3390/jpm11090852/s1, Table S1: hazard ratios for the association between CBR expression and survival with adjustment for prognostic factors.
