# Supplementary material for: Association of Breast Tumour Expression of Cannabinoid Receptors CBR1 and CBR2 with Prognostic Factors and Survival in Breast Cancer Patients
Source: J Pers Med. 2021 Aug 28;11(9):852. doi: 10.3390/jpm11090852 (PMC8466730; doi:10.3390/jpm11090852)
Supplement: Supplementary file 1 [file jpm-11-00852-s001.zip › jpm-1308128-supplementary.pdf]

Supplemental material for: Association of breast tumour expression of cannabinoid receptors CBR1 and CBR2 with prognostic factors and survival in breast cancer patients

Authors: Jessica Morin-Buote[1,2,3,†], Kaoutar Ennour-Idrissi[1,2,3,4,†], Éric Poirier[2,5,6], Julie Lemieux[2,6,7], Daniela Furrer[2], Anna Burguin[2,3,8], Francine Durocher[2,3,8] and Caroline Diorio[1,2,3,6,*]

[1] Department of Social and Preventive Medicine, Faculty of Medicine, Laval University, Quebec, QC, Canada

[2] CHU de Québec-Université Laval Research Center, Quebec, QC, Canada

[3] Université Laval Cancer Research Center, Quebec, QC, Canada

[4] Department of Molecular Biology, Medical Chemistry and Pathology, Faculty of Medicine, Laval University, Quebec, Qc, Canada

[5] Department of Surgery, Faculty of Medicine, Laval University, Quebec, QC, Canada

[6] Centre des Maladies du Sein, Hôpital Saint-Sacrement, Quebec, QC, Canada

[7] Department of Medicine, Faculty of Medicine, Laval University, Quebec, QC, Canada

[8] Department of Molecular Medicine, Faculty of Medicine, Laval University, Quebec, QC, Canada

[*] Correspondence: caroline.diorio@crchudequebec.ulaval.ca; Tel 1 418-682-7511 #84726

[†] Equally contributed as first authors.

TABLE S1. HAZARD RATIOS FOR THE ASSOCIATION BETWEEN CBR AND SURVIVAL WITH ADJUSTMENT FOR PROGNOSTIC FACTORS

Table S1. Hazard ratios for the association between CBR and survival with adjustment for prognostic factors

CBR	Events/total	Crude HR (95% CI)		p-value	Adjusted§ HR (95% CI)		p-value	Fully adjusted† HR (95% CI)		p-value
**Overall survival**										
CBR1	81/489	0.98	[0.88-1.09]	0.70	1.00	[0.89-1.12]	1.00	1.01	[0.90-1.12]	0.87
CBR2 cytoplasmic	82/475	0.95	[0.82-1.09]	0.46	1.02	[0.87-1.19]	0.82	1.03	[0.87-1.21]	0.76
CBR2 nuclear	82/475	0.99	[0.90-1.09]	0.76	1.04	[0.94-1.16]	0.44	1.03	[0.93-1.15]	0.54
**Recurrence-free survival**										
CBR1	67/489	1.04	[0.93-1.17]	0.49	0.95	[0.83-1.08]	0.42	0.98	[0.86-1.12]	0.77
CBR2 cytoplasmic	68/475	0.96	[0.83-1.11]	0.59	1.12	[0.95-1.33]	0.19	1.16	[0.96-1.39]	0.12
CBR2 nuclear	68/475	0.99	[0.90-1.09]	0.77	1.04	[0.92-1.16]	0.62	1.04	[0.92-1.17]	0.56
**Event-free survival**										
CBR1	134/489	1.00	[0.92-1.09]	0.97	0.97	[0.88-1.06]	0.46	0.97	[0.89-1.06]	0.50
CBR2 cytoplasmic	134/475	0.98	[0.87-1.10]	0.71	1.01	[0.89-1.14]	0.90	0.99	[0.87-1.12]	0.88
CBR2 nuclear	134/475	0.98	[0.91-1.06]	0.69	1.00	[1.00-1.00]	0.44	1.00	[1.00-1.00]	0.24

CBR: cannabinoid receptor, CBR1: cannabinoid receptor 1; CBR2: cannabinoid receptor 2; HR: hazard ratio; CI: confident interval; § Model included age, menopausal status, family history of breast cancer, smoking status, alcohol consumption, personal history of breast cancer, neoadjuvant chemiotherapy, neoadjuvant endocrine therapy, tumour size, stage, tumour grade, lymph node involvment, ER/PR status, HER2 status; † Model included age, menopausal status, family history of breast cancer, smoking status, alcohol consumption, personal history of breast cancer, neoadjuvant chemiotherapy, neoadjuvant endocrine therapy, tumour size, stage, tumour grade, lymph node involvment, ER/PR status, HER2 status, year diagnosis, adjuvant radiotherapy, adjuvant endocrine therapy, anti-HER2 therapy,type of surgery.
